# Comparison of Posterior Retroperitoneoscopic Adrenalectomy Versus Lateral Transperitoneal Laparoscopic Adrenalectomy for Adrenal Tumors: A Systematic Review and Meta-Analysis

**DOI:** 10.3389/fonc.2021.667985

**Published:** 2021-05-10

**Authors:** Chunyang Meng, Chunxiao Du, Lei Peng, Jinze Li, Jinming Li, Yunxiang Li, Ji Wu

**Affiliations:** ^1^ Department of Urology, Nanchong Central Hospital, The Second Clinical Medical College, North Sichuan Medical College (University), Nanchong, China; ^2^ Department of Clinical Pharmacy, Sichuan Cancer Hospital and Institute, Sichuan Cancer Center, School of Medicine, University of Electronic Science and Technology of China, Chengdu, China; ^3^ Department of Urology, The Affiliated Hospital of Medical College, North Sichuan Medical College (University), Nanchong, China

**Keywords:** adrenal tumor, adrenalectomy, posterior retroperitoneoscopic adrenalectomy, lateral transperitoneal adrenalectomy, meta-analysis

## Abstract

**Objective:**

To discuss the differences in the effectiveness and security for adrenal tumors by posterior retroperitoneoscopic adrenalectomy (PRA) and lateral transperitoneal laparoscopic adrenalectomy (LTA).

**Methods:**

We systematically searched PubMed, Embase, Scopus database and Cochrane Library, and the date was from above database establishment to November 2020. Stata 16 was used for calculation and statistical analyses.

**Results:**

Nine studies involving eight hundred patients were included. The following differences were observed in favor of PRA vs LTA: less operative time (MD: −22.5; 95% CI −32.57 to −12.45; P=0.000), Fewer estimated blood loss (MD: −15.17; 95% CI −26.63 to −3.72; P=0.009), lower intensity of postoperative pain (MD: −0.56; 95% CI, −1.05 to −0.07; P=0.026), shorter length of hospital stay (MD: −1.15; 95% CI −1.94 to −0.36; P=0.04). No differences were shown in conversion rate (OR 2.07; 95%CI 0.71 to 6.03; P=0.181) and complications (OR 0.85;95% CI 0.46 to 1.56; P=0.597).

**Conclusions:**

Posterior retroperitoneoscopic adrenalectomy was clinically superior to lateral transperitoneal laparoscopic adrenalectomy for adrenal tumors in operative time, estimated blood loss, length of hospital stay, and postoperative pain. Only in term of conversion rate and complications, both were similar

## Introduction

Since the adrenalectomy successfully performed for the first time, adrenal surgery has seen great advances, especially in terms of minimally invasive surgery. First study of laparoscopic adrenalectomy (LA) was published in 1992 (1). Nowadays LA was developed into the “gold standard” technique to treat small to medium size adrenal tumors. Compared with open adrenalectomy, the main advantages of LA are short hospitalization, low morbidity rate, and a rapid recovery (2, 3). Among the multiple approaches of LA, lateral transperitoneal adrenalectomy (LTA) was more popular, because most surgeons were more familiar with anatomy and operating view. However, posterior retroperitoneoscopic adrenalectomy (PRA) has been increasingly favored by urologists in recent years for the reason that it could directly and rapidly get into the surgical area without mobilizing intraperitoneal organs. Moreover, PRA has an outstanding advantage, which could accomplish bilateral adrenalectomy without repositioning the patient (4).

Nevertheless, up to present, the final choice between RPA or LTA still remains influenced by surgeon’s preference (5). The advantages of two surgical access during laparoscopic has not been clearly highlighted yet. Although several comparative studies of the two techniques had been published, most were retrospective studies and controversial.

In order to fill the gap, we conducted the meta-analysis to evaluate and compare the perioperative outcomes in patients treated with PRA and LTA for adrenal tumor.

## Methods

### Literature Search and Eligible Criteria

We systematic searched PubMed, Embase, Scopus database and Cochrane Library databases to identify studies from the date of database establishment to November 2020. Search terms included: “adrenal tumor”, “posterior retroperitoneoscopic adrenalectomy”, “lateral transperitoneal adrenalectomy”, “retroperitoneoscopic”, “lateral transperitoneal”, “adrenalectomy”, and the search was not restricted by language. Meanwhile, we also performed manual retrieval from the references of subject-related studies to broaden the search.

Studies meeting the following inclusion criteria were admitted: (1) patients diagnosed as unilateral adrenal tumor by urologist; (2) studies should explicitly describe their techniques as PRA or LTA, and make a comparison; (3) full papers containing at least one outcome parameters, such as operative time, estimated blood loss, postoperative pain, length of hospital stay, conversion rate and complications; (4) research types should be not only randomized controlled study but also prospective controlled study and retrospective study. The following studies were excluded: reviews, case reports, letters, low-quality researches and researches with no detailed data.

two investigators (CX.D and L.P) completed this process independently, and differences between investigators were settled by consultation. The third reviewer (YX.L) was involved in the judgment if an agreement could not be reached.

### Data Extraction

The following data from each study would be extracted into our meta-analysis: lead author, publication date, study type, study country, study interval, sample size, operation time (OT), estimated blood loss (EBL), length of hospital stay (LOS), conversion rate, postoperative pain at day one and perioperative complications. When continuous variables reported as other forms in the main literature, we calculated the mean and standard deviation (6).

### Risk of Bias Assessments

Not only using ROBINS-I tool for non-randomized studies but also using Cochrane’s criteria for RCTs to evaluate Publication bias. ROBINS-I tool included seven domains: confounding bias, selection bias, bias in measurement classification of interventions, bias due to deviations from intended interventions, bias due to missing data, bias in measurement of outcomes, and bias in selection of the reported result (7). Cochrane’s criteria contained random sequence generation, allocation concealment, blinding of participants and personnel, blinding of outcome assessment, incomplete outcome data, selective reporting and other bias (8).

### Study Quality Assessment

We used the Jadad scale (9) to assess randomized controlled trials (RCT) and the NOS scoring rules (10) for non-randomized controlled studies. The Jadad score ranged from zero to seven points, and the quality score more than four points was ranked as high quality. NOS scale was nine stars in all, and the study of more than six stars was divided into high quality.

### Data Analysis

We performed using Stata 16 for data analysis, and made use of mean difference (MD) and odds ratio (OR) to evaluate the continuous and dichotomous outcomes, respectively. The 95% confidence interval (CI) and P value should be counted. I-square tests were used to verify the heterogeneity between the included studies. In addition, sensitivity analysis should be done to interpret potential source of heterogeneity if the heterogeneity more than fifty percent.

### Registration

This study registered on PROSPERO and registration number was: CRD42021230470.

## Results

### Description of Studies

**Figure 1:**
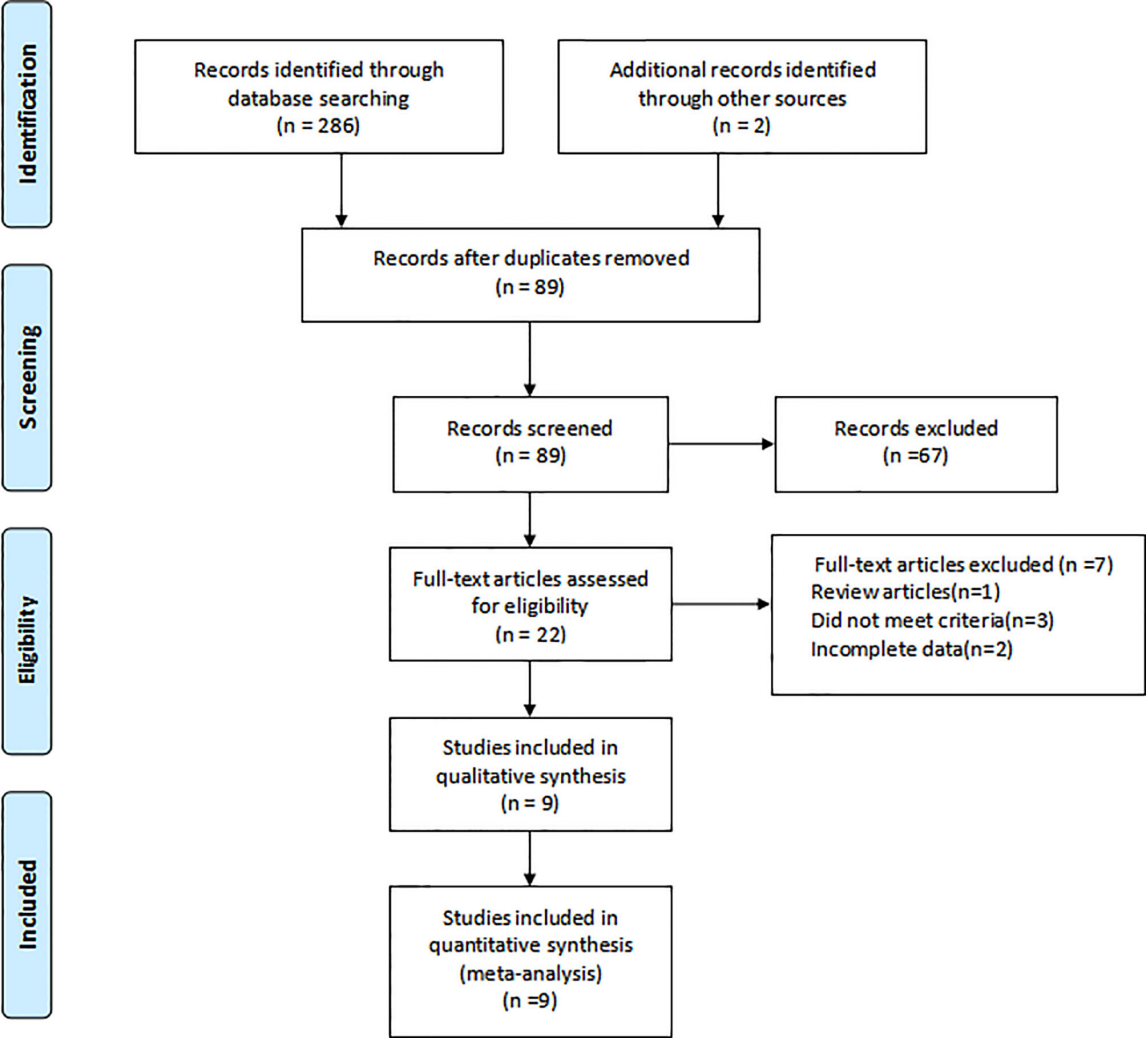
Flow diagram of studies selection process.

In all, 286 researches were identified, and nine studies of them were eventually determined. The screening process was shown in **Figure 1**. The characteristics of the included studies were summarized in **Table 1**. The nine included studies were published between 2002 and 2019, total including 800 patients. Furthermore, included studies’ sample size was in the 46 to 159 range. Among them, four were retrospective design, two were prospective design, and three were randomized controlled trials.

**Table 1:** Baseline characteristic of included studies.

Studies (year)	Country	Interval	Intervention	No. patients	Age (years)	BMI (kg/m^2^)	Tumor size (cm)	Clinical/pathological Diagnosis PRA/LTA	Study design	Quality score
Barczynski (11)	Poland	January 2006 to June 2008	PRA	33	47.9(43.5-52.2)^a^	27.6 (26.1-29.0)^a^	39.3 (33.3-45.3)^ac^	PHA:7/7	RCT	4
			LTA	32	46.6(41.6-51.6)^a^	27.3 (25.9-28.8)^a^	40.3 (34.5-46.1)^ac^	Cushing’s:4/3		
								PCC:8/7		
								NFA:14/15		
Ban (12)	Korea	January 2008 to March 2015	PRA	31	48.74 ± 13.9	22.64 ± 2.5	3.77 ± 1.64	PCC:31/22	Retrospective	7
			LTA	22	47.32 ± 14.17	23.92 ± 4.74	6.24 ± 2.84			
Chai (13)	Korea	September 2012 to February 2016	PRA	41	46.4 ± 11.0	23.6 ± 3.0	3.0 ± 1.3	PHA:16/20	RCT	5
			LTA	42	48.0 ± 11.4	24.2 ± 3.3	2.9 ± 1.4	Cushing’s:10/7		
								PCC:7/8		
								NFA:8/7		
Berber (14)	U.S.A	1994 to 2008	PRA	90	51 ± 14	NA	NA	PHA:31/3	Retrospective	7
			LTA	69	52 ± 14			Cushing’s:15/9		
								PCC:10/7		
								NFA:20/18		
								Other:12/16		
Dickson (15)	U.S.A	May 2000 to December 2009	PRA	23	47.3 ± 16.1	26.2 ± 6.6	3.3 ± 1.8	PCC:23/23	Retrospective	6
			LTA	23	42.0 ± 18.1	26.1 ± 5.4	4.0 ± 2.2			
Kozlowski (16)	Poland	February 2015 to June 2018	PRA	44	59.3 ± 10.2	29.1 ± 5.2	4.0(0.8-7.5)^b^	PCC:9/4	RCT	5
			LTA	33	61.2 ± 8.3	30.1 ± 6	4.1(1.5-7.5)^b^	Cushing’s:3/5		
								PHA:4/2		
								NFA:28/22		
Lezoche (17)	Italy	1994 to 1998	PRA	67	45.9(17-84)^b^	NA	3.0(2.5-7)^b^	PHA:20/23	Prospective	6
			LTA	72	46.3(23-74)^b^		4.7(3-11)^b^	Cushing’s:29/15		
								PCC:13/8		
								NFA:5/26		
Lombardi (18)	Italy	1999 to 2007	PRA	38	48.9 ± 11.9	NA	30.9 ± 9.6^c^	NFA:32/31	Retrospective	6
			LTA	38	45.2 ± 13.0		32.9 ± 12.1^c^	PCC:6/7		
Van Uitert (19)	Netherlands	February 2007 to December 2014	PRA	64	51 ± 13	28 ± 5	2.8 ± 2.2	NA	Prospective	7
			LTA	38						

PRA, posterior retroperitoneoscopic adrenalectomy; LTA, lateral transperitoneal laparoscopic adrenalectomy; NA, not available; RCT, randomized controlled trial; PHA, primary hyperaldosteronism; PCC, phaeochromocytoma; NFA, non-functioning adenoma; Cushing’s, ACTH-independent adrenal disease unless otherwise stated.

^a^mean (95%CI); ^b^median (range); ^c^the unit of length is millimeter.

### Quality Assessment

The quality of all included RCTs was relatively high (Jadad scale: 4, 5 and 5 of 7 points). Based on NOS scale, three literature was scored as six stars and three as seven stars. The median of the NOS Score was 7. We also had provided the final quality assessment list (**Table 1**).

### Perioperative Outcomes

#### Operative Time

**Figure 2:**
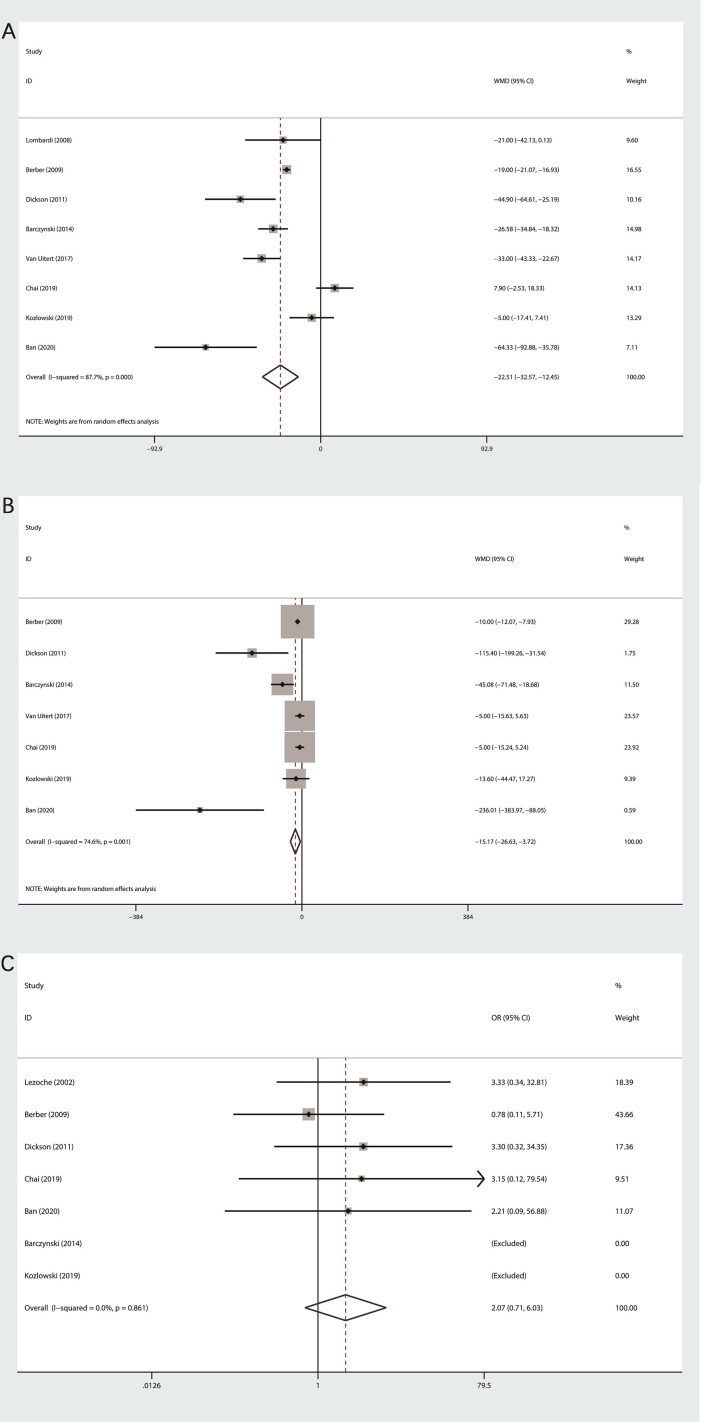
Forest plot of perioperative outcomes between PRA and LTA. **(A)** Operation time. **(B)** Estimated blood loss. **(C)** Conversion rate.

Data about OT were extracted from eight studies (11–16, 18, 19),with high heterogeneity among studies (I^2^>50%), so we used random effect mode. Our final meta-analysis indicated that PRA group was less than LTA group in terms of OT (MD: −22.5; 95% CI −32.57 to −12.45; P=0.000, **Figure 2A**).

#### Estimated Blood Loss

Among the seven studies on the EBL (11–16, 19), obvious heterogeneity was observed, so we applied random effect model to statistical analysis. Our final outcomes indicated statistically significant difference between PRA and LTA (MD: −15.17; 95% CI, −26.63 to −3.72; P = 0.009, **Figure 2B**).

#### Conversion Rate

The conversion rate was recorded in seven of nine researches (11–17). Since the heterogeneity test outcome (I^2^ = 0%), we used fixed effects model. No statistical differences were observed between the two group (OR 2.07; 95%CI 0.71 to 6.03; P=0.181, **Figure 2C**).

### Postoperative Indicators

#### Length of Hospital Stay

**Figure 3:**
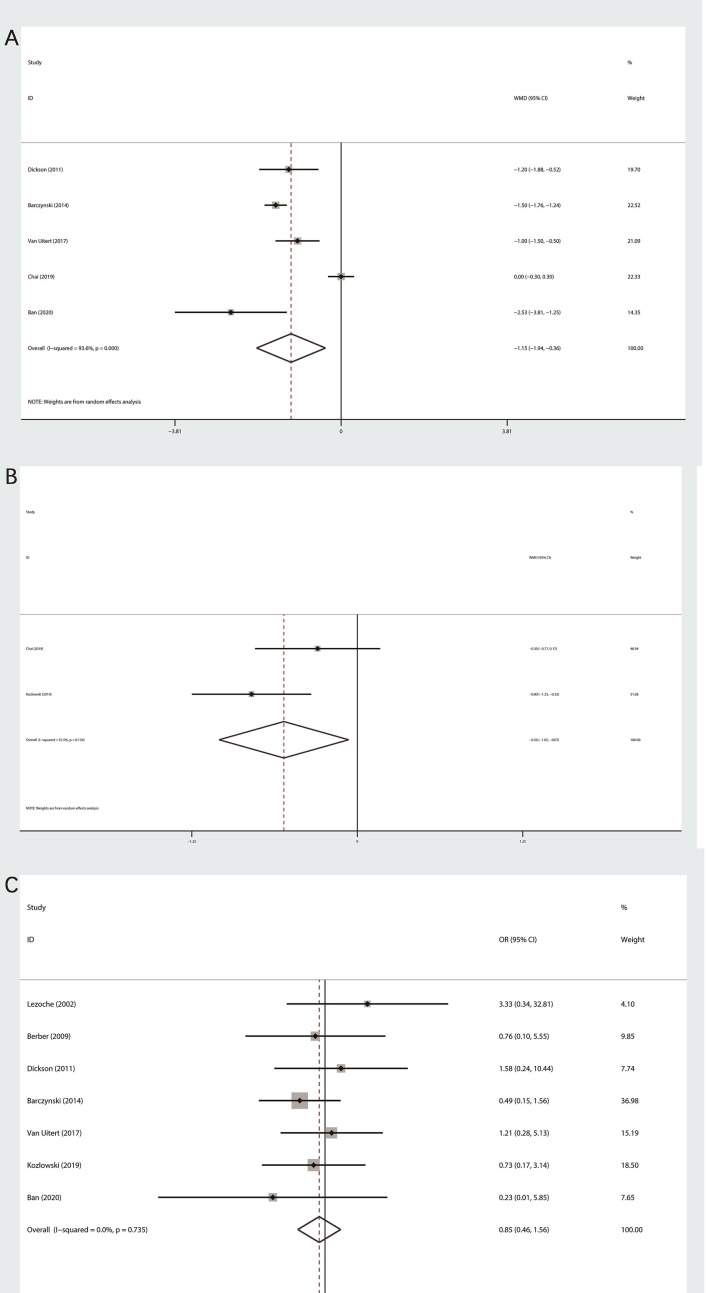
Forest plot of postoperative outcomes between PRA and LTA. **(A)** Length of hospital stay. **(B)** Postoperative pain at day one. **(C)** Complication.

Five studies, which consist of 349 patients, were related to LOS (11–13, 15, 19). The random effect model was used according to this outcome of heterogeneity test (I^2^>50%). Last result showed that PRA group was less than LTA group in terms of LOS (MD: −1.15; 95% CI −1.94 to −0.36; P=0.04, **Figure 3A**).

#### Postoperative Pain at Day 1

A total of two studies related to postoperative pain at day one (13, 16), which were evaluated by visual analog scale (from 1 to 10). We performed a random effect model, because the heterogeneity was significant (I^2^ = 55.5%). The final outcome indicated that patients in PRA group subjectively believed that lower intensity of postoperative pain (MD: −0.56; 95% CI −1.05 to −0.07; P=0.026, **Figure 3B**).

#### Perioperative Complications

We had done meta-analysis of perioperative complication (11, 12, 14–17, 19). Based on the heterogeneity test, fixed effect models were used. The final results indicated that there was no statistical significance among two surgical technique (OR 0.85;95% CI 0.46 to 1.56; P=0.597, **Figure 3C**).

### Sensitivity Analysis

**Figure 4:**
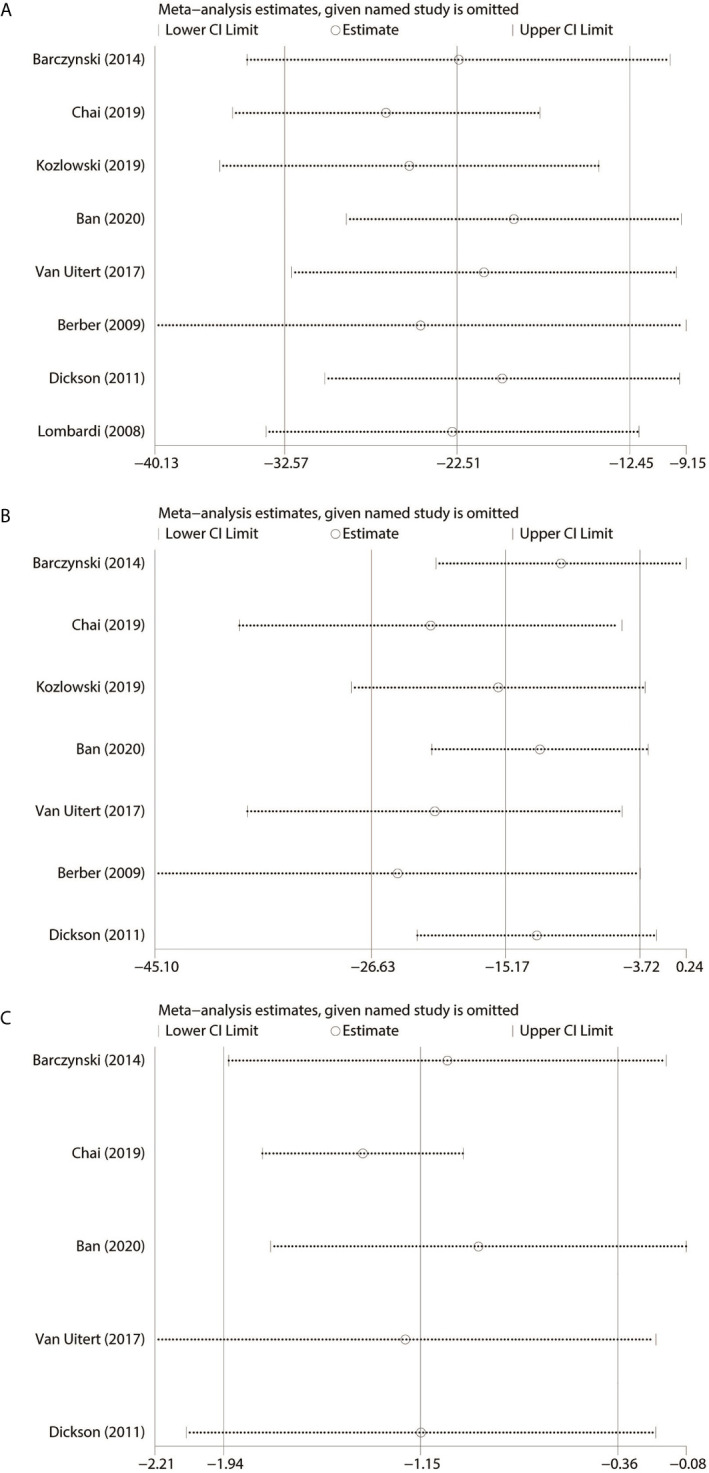
Sensitivity analysis. **(A)** Operating time. **(B)** Estimated blood loss. **(C)** Length of hospital stay.

Based on the outcomes of heterogeneity, such as OT, EBL and LOS, we tested the sensitivity of the results to improve the reliability of the analysis. Studies were removed one by one to recalculate the combined mean difference, and we found that results of our meta-analysis were stable except EBL (**Figure 4**).

#### Risk of Bias of Included RCTs

**Figure 5:**
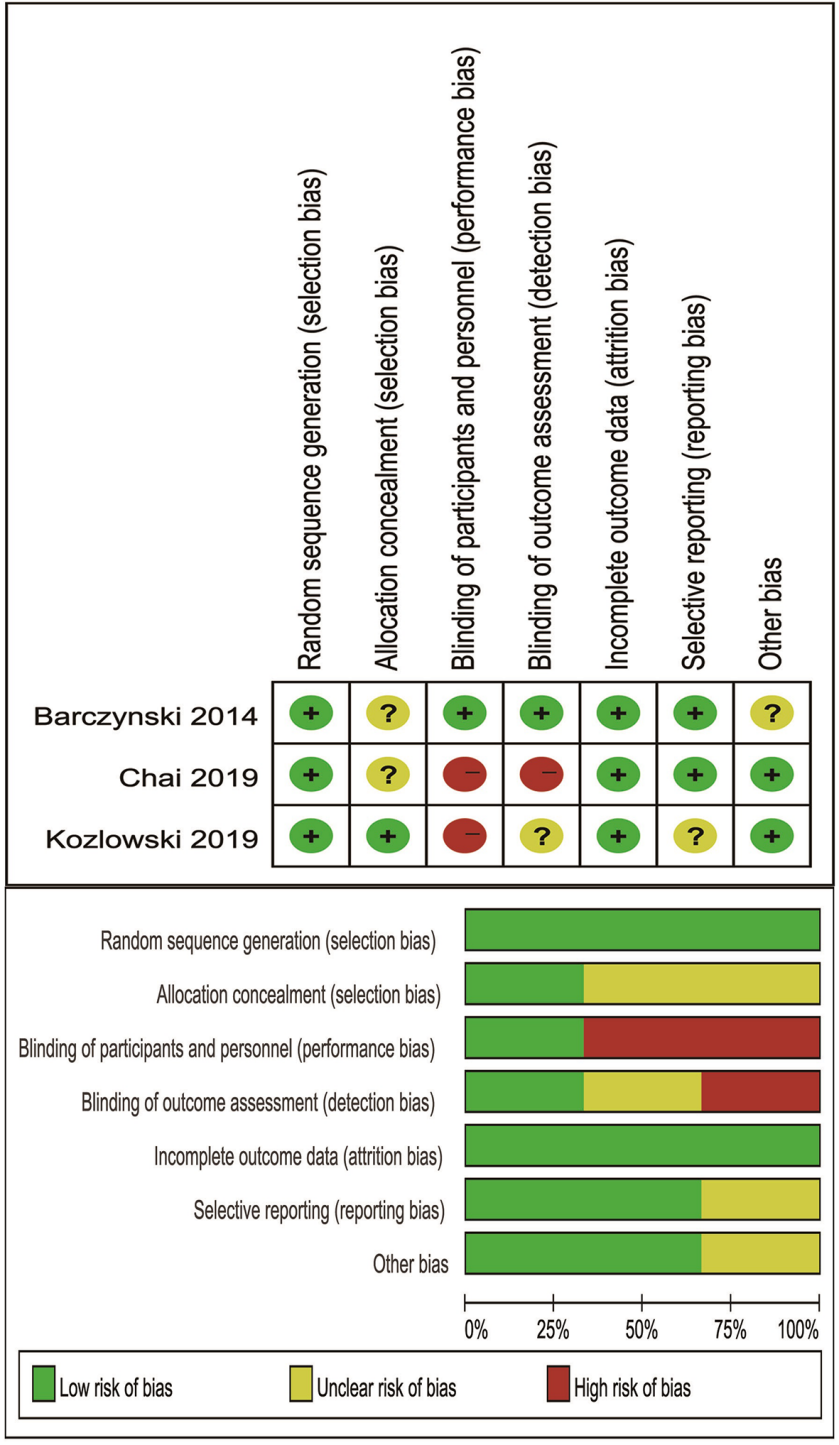
Risk of bias of included RCTs.

Cochrane’s criteria were performed to evaluate risk of bias, and major weakness was in the domains of blinding of participants and personnel. The final results were listed in **Figure 5**.

#### Risk of Bias of Included Non-Randomized Studies

The ROBINS-I tool was used to assess risk of bias, and final results suggested that all comparative studies had a moderate risk of bias. We found that the main weakness was in selection bias.

## Discussion

In the last decades, adrenalectomy has made a major breakthrough, which turned a large incision over to a minimally invasive. The dominant position of minimally invasive surgery had less operative time, reduced postoperative pain, and shorter hospital stay. Since LTA was proposed in 1992, its surgical indications had been widely increased (1, 20). At present, the technique is not only considered to be the “gold standard” procedure for adrenal tumors less than six cm in diameter around the world, but also could be performed by experienced surgeons to treat patients with larger tumor diameter (21, 22). On the contrary, PRA, popularized by Waltz, was an alternative method of adrenal tumors, especially for patients with a history of abdominal surgery (4). In addition, PRA has shown its feasible for adrenal tumors less than eight cm in diameter (16).

However, previous studies were mostly retrospective and were not sufficiently powered to demonstrate the superiority between the two procedures. In the meantime, the latest high-quality RCTs have been reported. Based on the above reasons, we performed this meta-analysis to discuss the differences in the effectiveness and security for adrenal tumors by two surgical method.

In terms of OT, our meta-analysis showed that PRA group was less than LTA group, and it was consistent with most studies. It could directly and rapidly get into the adrenal gland by PRA, which means taking less time during dissection to separate tissues for skilled surgeons. However, the research performed by Kozłowski et al. (16) and Chai et al. (13) both found no significant difference between the two methods with respect of OT. In the professor Kozłowski’s study, we found the main reason may be study subjects with morbid obesity or high BMI, making perirenal fat dissection extremely difficult, even though the surgeon has ten years of experiences in laparoscopic adrenalectomies. It has been proven that high BMI was correlate significantly with the duration of surgery (23). In the professor Chai’s study, the surgeon was lack of experience of PRA (only 29 cases) at the start of the study, which indicated far from reaching proficiency (19, 23). Moreover, different categories of adrenal tumors, male to female sex ratios, tumors location, definitions of surgical time were also interfering factors (24).

Our meta-analysis indicated that PRA group was less than LTA group with regard to EBL, and the same in LOS. In a sense, the outcomes indicated that patients of the PRA group suffered less tissue damage during the operation and had faster recovery after surgery, which may be caused by anatomic structure and gas pressure. The significant difference in EBL was possibly caused by relatively less dissection during PRA. As is known to all, the less the anatomical separation, the relative reduction in blood loss. Furthermore, during PRA, higher gas insufflation pressure (usually more 20 mm Hg) was used to induce tamponade of minor bleeding from small vessels. Meanwhile, the high retroperitoneal insufflation pressure usually could lead to compress the IVC or renal vein, reduce venous returns from the adrenal gland and ultimately reduce intraoperative blood loss (12). LOS was an important indicator to evaluate the postoperative recovery. In addition, shorter LOS also meant less hospitalization medical expenses of patients to a certain extent, and relatively higher turnover rate of hospitals.

Based on patient autonomy score by the visual analogue scale (VAS), the final Meta-analysis had evaluated the postoperative pain at day one, and demonstrated that there was statistical significance between two groups. It can be obviously observed that acetaminophen (65mg, tid) was used for postoperative analgesia in professor chai’s study (13). On the other hand, professor Kozłowski and his colleagues performed postoperative analgesia by instilling bupivacaine solution (10ml,0.5%) to the skin and subfascial space prior to skin incisions (16). A Cochrane meta-analysis on 75 studies of acetaminophen found its analgesic effect decreased after 6 h (25). Unfortunately, there is not yet high-quality evidence on the superiority of these two modalities in postoperative pain management. Due to the small number of studies and choice of postoperative analgesic management, we were cautious about this conclusion. Besides, the degree of patient tolerance to pain may also be a contributing factor.

Concerning complication rate, no statistical difference was observed between PRA and LTA groups (OR 0.85;95% CI 0.46 to 1.56; P=0.597, **Figure 3C**). Similarly, a network meta-analysis about adrenalectomy also had shown that no statistical difference was found (26). Additional, Jiang et al. performed a meta-analysis about pheochromocytoma and reported a similar result (OR: 1.58, 95% CI: 0.58 to 4.33, p > 0.05) (27).

A largest matched pair multi-institutional study in minimally invasive partial nephrectomy, showed that retroperitoneal approach seemed to have a slighter intraoperative complication rate, longer operative times and earlier postoperative recovery when compared with transperitoneal (28). Although the approach was not exactly the same, it was also confirmed from the side that retroperitoneal approach was better in the overall curative effect of urological surgery. This was similar to our conclusion.

A previous study showed that robot-assisted laparoscopic radical nephrectomy with adrenalectomy might be a safe procedure for adrenal compromise (29). For the purpose of to facilitate tumor removal, or due to renal pedicle injury during adrenalectomy, concomitant nephrectomy also was an option. In some special cases, such as patients with congenital solitary kidney or patients with poor contralateral renal function, it was not appropriate. Strengths and weaknesses of these two approaches in laparoscopic adrenalectomy with nephrectomy remains to be demonstrated by evidence-based medicine.

3D laparoscopy was important for urological surgeries, since it could improve the depth of perception and lead to better visibility. A meta-analysis including 17 studies showed 3D laparoscopy seemed to provide better clinical and surgical outcomes in some complex urological surgeries compared with conventional 2D laparoscopy (30). For a relatively easy adrenalectomy, the largest case-control study to date to our knowledge showed that 3D laparoscopy offered advantages over 2D laparoscopy, especially in operative blood loss and operative time (31). Existing studies seemed to show that 3D laparoscopy was one of the directions in the future. However, compared with robot-assisted laparoscopy, further researches are needed to prove which is better.

We completed this meta-analysis under the guidance of PRISM strictly (32), but several limitations remained. First, the included studies were not all randomized controlled trials and the patient size ended up with 800, which meant a lack of evidence. In particular, only two randomized controlled trials were available for postoperative pain at 1st post-operative day. Second, limited number of clinical studies included and fail to perform subgroup analysis based on confounding factors such as tumor classification and tumor location. Third, in the field of comparison between laparoscopic and robotic approach, there was a lack of comparative research on these two surgical approaches, and it was a significant limitation of our study. Fourth, significant heterogeneity exists in some evaluation indexes. After carefully reading the studies included, we find that heterogeneity may be caused by differences of medical institutions, the proficiency of surgeons, and statistical methods. Unfortunately, we cannot get the detailed information. Based on the above reasons, we conducted a sensitivity analysis.

## Conclusion

Posterior retroperitoneoscopic adrenalectomy was clinically superior to lateral transperitoneal laparoscopic adrenalectomy for adrenal tumors. Only in term of conversion rate and complications, both operative methodologies were similar. more studies are still required to support our conclusion, especially in the management of postoperative pain.

## Data Availability Statement

The data sets presented in this study can be found in online repositories. The names of the repository/repositories and accession number(s) can be found in the article/supplementary material.

## Author Contributions

Conceived and designed the experiments: YL and JW. Analyzed the data: CM, LP, and CD. Contributed reagents/materials/analysis: JZL, JML, and JW. Wrote the manuscript: CM and LP. All authors contributed to the article and approved the submitted version.

## Funding

This study was supported by Sichuan Science and Technology Program under Grant number 2020YFS0320.

## Conflict of Interest

The authors declare that the research was conducted in the absence of any commercial or financial relationships that could be construed as a potential conflict of interest.
